# Study on Electrical Performance of a U-Type Microfluidic Acceleration Switch Using Salt Solution as the Sensitive Electrode

**DOI:** 10.3390/s20247062

**Published:** 2020-12-10

**Authors:** Teng Shen, Yang Chen, Jiaqing Chang, Jianhui Zhang, Xingxing Liu

**Affiliations:** 1 School of Mechanical and Electrical Engineering, Guangzhou University, Guangzhou 510006, China; shent215@gzhu.edu.cn (T.S.); jqchang@gzhu.edu.cn (J.C.); zhangjh@nuaa.edu.cn (J.Z.); 2 School of Mechanical, Fuzhou University, Fuzhou 350108, China; liuxingxing432@fzu.edu.cn

**Keywords:** inertial switch, dynamic threshold, salt solution, anti-high overload

## Abstract

The threshold of microfluidic inertial switch is excessively dependent on the size of the passive valve structure and the gas–liquid surface energy of working liquid. How to achieve high threshold and anti-high overload using liquid with low viscosity and low surface tension is a challenging work. Based on the designed U-type microfluidic inertial switch, the electrical characteristic of salt solution at microscale as well as the threshold and dynamic electrical performance of switch were studied. The VOF and CSD modules in CFD software were employed to analyze the dynamic flow process, and then the air–liquid surface moving displacement curve was compared by the theoretical model. A self-designed acceleration test platform was utilized to measure the static threshold, dynamic threshold, and anti-high overload of the inertial switch. The results show that the U-type microfluidics inertial switch using salt solution as sensitive electrode has better performance in power connection and anti-high overload. In particular, it also has the ability to achieve a range of dynamic threshold by changing the placement of the contact electrode, which can achieve rapid power on and off.

## 1. Introduction

Due to well-known advantages of fast response compactness, low-cost production, better overall process control, and low sample consumption, the microfluidics sensing method is widely used in the field of biochemical analyses, environmental monitoring, food safety, and body health. On the whole, the microfluidics sensing and detection technologies can be divided into two categories: (1) microfluidics as the analysis source to realize the detection by analyzing the characteristic elements in the sample [1,2,3,4,5]; and (2) microfluidics as sensing elements to detect external sensitive sources, such as acceleration, pressure, and temperature, by analyzing the corresponding flow behaviors. For example, a novel design for a tilt sensor utilizing the movement of an excited ferrofluid was presented by DeGraff [6]. Davis [7] reported a microfluidics temperature sensor using a noninteracting technique based on surface plasmon resonance interrogation. Joo et al. [8] presented a novel triple-state liquid-based resistive microfluidic tactile sensor with high flexibility, durability, and sensitivity. Kim et al. [9] developed a digital microelectromechanical system (MEMS) accelerometer consisting of a microscale liquid metal droplet in a microchannel etched on photosensitive glass.

Inertial switches based on MEMS are widely used in different fields such as airbags, crash recorders, and arming systems [10,11]. According to the contact mode, the MEMS inertial switches can be divided into solid-to-solid type and liquid-to-solid type. Generally, the solid-to-solid electrical contacts, which use solid proof masses and beams, are susceptible to arcing, contact erosion, adhesion, micro welding, and oxidation, resulting in increasing the contact resistance dramatically [12,13]. By comparison, the microfluidics inertial switch is a typical representative of using microdroplet as sensing element, which can avoid the problems of signal bouncing and contact wear during the switching motion [14]. Therefore, this research field has received extensive attention in recent years. Examples include the time-delay switch integrating with microfluidic system by Huang [15], the automatic-recovery microfluidics inertial switch by Shen [16], the MEMS inertial switch using microscale liquid-metal (LM) droplet by Yoo [17], and the passive inertial switch using MWCNT–hydrogel by Kuo [18]. Although the existing microfluidic inertial switches are characterized by sensitive response, simple structure, and low price, due to the limitation of valve structure [19,20] and droplet surface performance [15,21], the threshold value is fixed and concentrated from dozens to a hundred g (acceleration parameter). Moreover, microfluidic inertia switches usually employ metal droplets as moving electrodes, such as mercury and EGaIn, which have high surface tension and hydrophobic performance, but are not easily controlled in volume [22] and are easily oxidized [23]. Therefore, how to achieve high threshold and anti-high overload using liquid with low surface tension is a challenging work at the moment.

**Figure 1:**
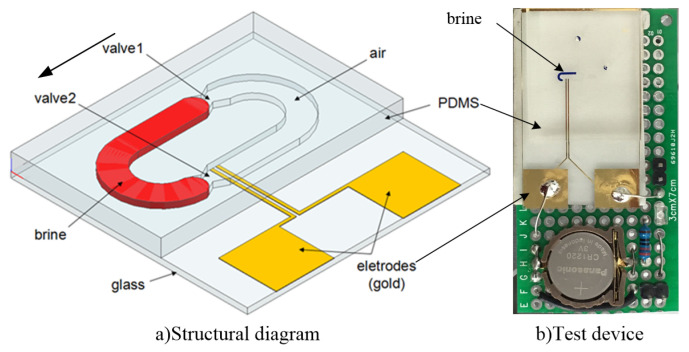
The designed U-type microfluidics inertial switch.

Therefore, to solve the above problems, we explore the design method of microfluidics inertial switch in high overload environment [24], and the structure and principle analysis of a U-type microfluidic inertial switch has been studied before [25]. The three-dimensional structure including glass cover-plate, PDMS substrate, contact electrode, U-type microchannel, and the sensitive electrode (salt solution) and the test device which is integrated into the microcircuit board are shown in Figure 1. The metal electrode is deposited on the side of the glass cover-plate that is bonded to the PDMS substrate, and the external circuit can be turned on when the salt solution breaks through Capillary Valve 2 to connect the metal electrode. When the acceleration load is small, the microfluid is blocked by Capillary Valves 1 and 2, so the switch will not turn on. Then, the liquid will flow through Capillary Valve 2 and contact with the electrode when the inertial load is large enough to overcome the resistance valve threshold. However, no matter how large the inertial load is, the liquid surfaces on both sides will be at the same height position due to gravity.

In this paper, based on the above microfluidics inertial switch, the dynamic flow characteristic of salt solution under different acceleration loads is firstly studied by CFD simulation (Computational Fluid Dynamics). Then, the electrical property of salt solution at microscale is measured using a glass capillary in visual resistance test platform. Finally, the proposed device with different structure sizes is tested to obtain the electrical characteristics and threshold characteristics. In summary, the main purpose of this research is to explore the feasibility of salt solution as sensitive electrode, as well as the threshold and power connection characteristics of the U-type microfluidic inertial switch.

## 2. Switch Dynamic Flow Simulation

**Figure 2:**
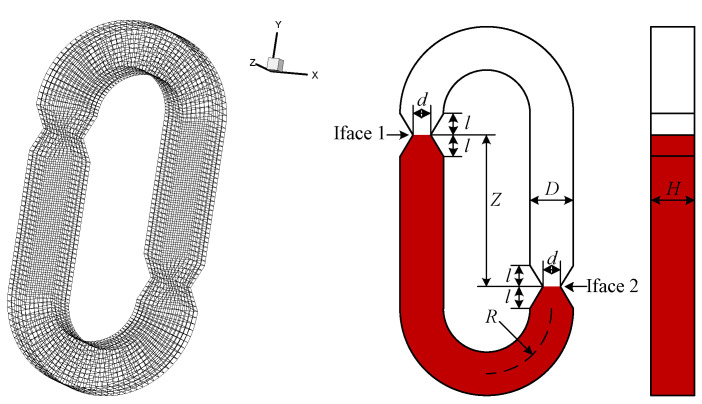
Diagram of the inertial switch structure model.

To understand the flow behavior in the U-type microchannel, Fluent VOF (Volume of Fluid) and CSF (Continuum Surface Force) modules were used to simulate the dynamic flow of the microswitch. Figure 2 shows the microchannel geometric model and grid division. The specific structural dimensions of the model are shown in Table 1, where *R*, *D*, *d*, H, l, and *Z* are the curved radius, width of the microchannel, width of the capillary passive valve, depth of the microchannel, half the length of the capillary passive valve, and the difference of liquid heights on both sides, respectively. The total number of grids divided in the simulation is 11,420, which verifies the grid independence. The simulated liquid uses salt solution, and the specific properties are shown in Table 1.

**Figure 3:**
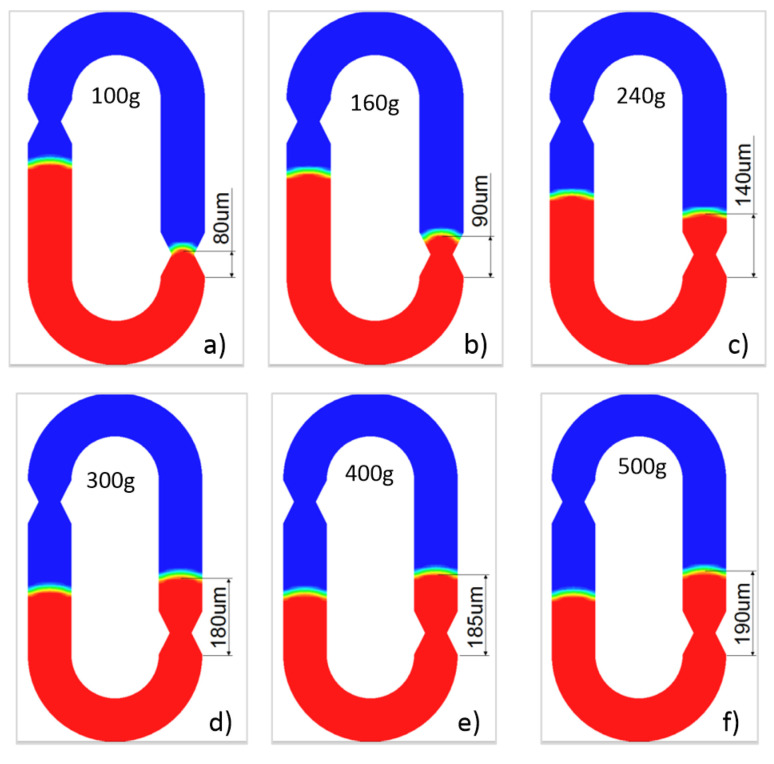
The distribution map. (**a**) under load of 100 g; (**b**) under load of 160 g; (**c**) under load of 240 g; (**d**) under load of 300 g; (**e**) under load of 400 g; (**f**) under load of 500 g.

A step-type acceleration signal with an amplitude of 100–500 g is applied in the sensitive direction of the switch (y negative direction) to obtain a dynamic flow cloud image of the moving electrode (brine). The distribution diagram of Gas–Liquid Interface 2 at the maximum movement displacement is shown in Figure 3. As the simulation results show, the switch can achieve a higher acceleration threshold and the moving liquid is continuous in the entire flow process, which indicates the U-shaped microchannel structure has the ability to resist gas–liquid separation, although the microfluid has low surface tension and low viscosity. In fact, even under the load of 3000 g, the liquid still does not appear to be dispersed.

**Figure 4:**
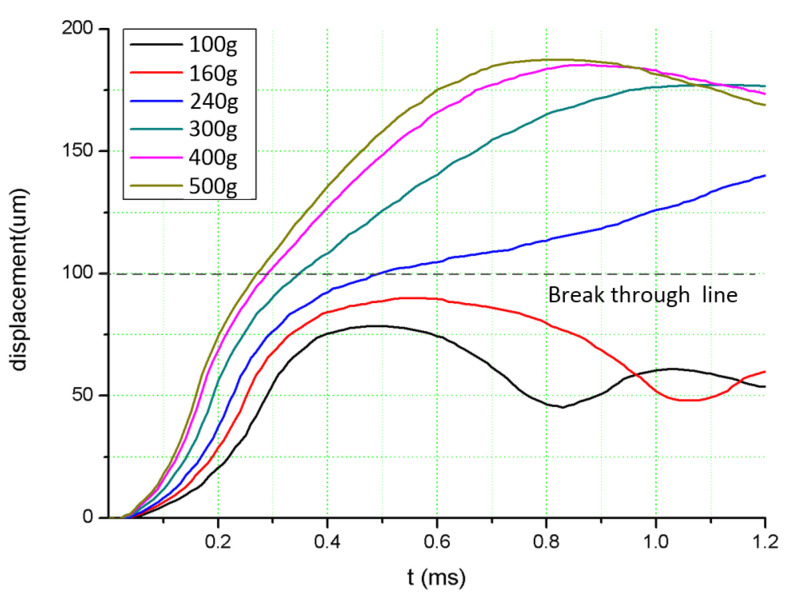
The simulation displacement-time curve of the switch.

Using TECPLOT software to post-process the simulation results, the time–displacement curve of Gas–Liquid Interface 2 is obtained, as shown in Figure 4. It can be seen that, when the acceleration amplitudes are 100 and 160 g, the displacement of Gas–Liquid Interface 2 is less than 100 µm, and the liquid surface is in a state of vibration, indicating that the liquid fails to pass through Capillary Valve 2, so the switch cannot be turned on. When the acceleration amplitude is 240, 300, 400, and 500 g, the curve passes through the 100-µm position, and the flow displacement shows a parabolic distribution, indicating that the liquid passes through Capillary Valve 2 and the switch is turned on. In addition, the comparison curve shows that, the greater is the acceleration load, the faster does the curve rise, indicating that the switch response time is shorter.

We studied the transient flow characteristics in the U-type microchannel previously [21], and the moving front time–displacement prediction model was established as follows:

(1) $$\frac{d^{2}z}{dt^{2}}+\frac{\mu }{C\rho }\frac{dz}{dt}+\frac{k}{2\left(z_{1}+z_{2}+R\pi \right)}{\left(\frac{dz}{dt}\right)}^{2}-\frac{a(z_{1}-z_{2})}{z_{1}+z_{2}+R\pi }=0$$

where *a*, *μ*, *k*, *C*, *ρ*, *R*, $z_{1}$, and $z_{2}$ are the acceleration, fluid viscosity, local resistance correction coefficient, microchannel feature parameter, fluid density, radius of the microchannel, and liquid surface heights at both ends, respectively. The above equation establishes the relationship between time, acceleration loads, and moving displacement.

**Figure 5:**
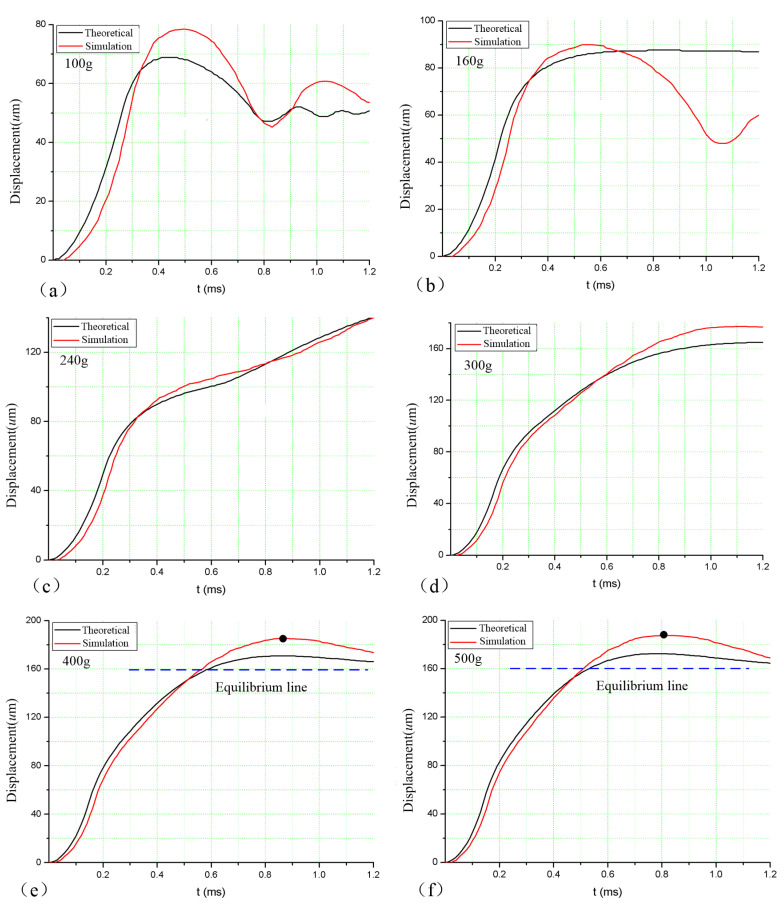
Comparison of theoretical and simulation curves. (**a**) under load of 100 g; (**b**) under load of 160 g; (**c**) under load of 240 g; (**d**) under load of 300 g; (**e**) under load of 400 g; (**f**) under load of 500 g.

Figure 5 shows the time–displacement comparison curve of the air–liquid surface simulation and the theoretical model. The simulation curve is the position change of the air–liquid surface with time, and the air–liquid phase line is 0.5. Overall, when the acceleration load is greater than the switching threshold, the theoretical curve and the simulation curve agree well, as shown in Figure 5c–f. However, when the acceleration amplitude is less than the switching threshold, the theoretical curve and the simulation curve agree well only in the initial ascending section, but the agreement after the extreme point is poor. This may be due to the fact that the cross-sectional shape factor of the microchannel does not account for the angle between the channel walls, and the theoretical model cannot accurately describe the oscillation of Gas–Liquid Interface 2 in the capillary passive valve. 

It is worth mentioning that, through high overload research, it was found that the U-shaped microchannel structure also has a dynamic threshold feature. As shown in Figure 5e,f, when the acceleration load is greater than the static threshold, the liquid surface moving state can be divided into three stages. (1) the liquid level displacement is greater than 100 µm, and the liquid breaks through the passive valve; (2) the liquid surface continues to move until it reaches the peak point, and the size of the peak point is related to the magnitude of the acceleration load (such as the peak points of 400 and 500 g are 165 and 168.5 µm, respectively); and (3) the acceleration load disappears, and the liquid surfaces at both ends of the U-shaped microchannel will end at the position of the balance line (160 µm) under the effect of their own gravity. Therefore, the dynamic threshold of the switch can be achieved by changing the placement of the contact electrode and using the characteristics of different peak displacements under different loads.

## 3. Switch Performance Experiment

**Figure 6:**
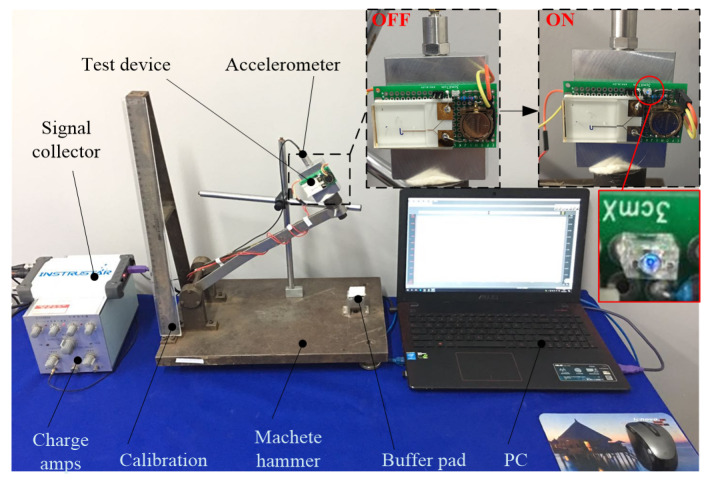
Custom-built machete hammer system.

We studied the conductivity of brine at microscale previously [21], and the resistivities of salt solutions with concentrations of 0.2, 0.6, and 1 g/mL are 0.077 Ω⋅m, 0.07 Ω⋅m and 0.051 Ω⋅m, which indicates that, in microscale space, salt solution still has better conductivity. To study the electrical performance of the encapsulated micro inertial switch, a small machete hammer was employed to produce the inertial acceleration signal, of which the size and pulse width can been adjusted by a cushion located at the point of fall. The measurement setup for acceleration threshold and electrical characteristics are shown as Figure 6 The proposed inertial switch is fixed on test board with a standard accelerometer (CA-YD-117) with sensitivity of 0.064 v/g, and a voltage division circuit with battery voltage (3 V) is integrated into the microswitch unit. During the measurement, the calibration is used to establish the corresponding relationship between the acceleration threshold and the height of the drop hammer.

Compared with the centrifugal experiment we did before, the impact acceleration experiment is more transient, so it is closer to the real load environment. Therefore, by gradually increasing the height of the drop hammer, the static acceleration thresholds of five switches with different structural size are obtained. When the acceleration load is larger than the static threshold, the LED in microswitch is lit, as shown in Figure 6. The comparison between the experimental value and the theoretical value is shown in Table 2. The theoretical threshold model was established in our previous research [25] as follows:

(2) $$a_{th}=\frac{\Delta p_{1}-\Delta p_{2}}{\rho (z_{1}-z_{2})}$$

where $\Delta p_{1}$ and $\Delta p_{2}$ are the capillary pressure at the end of Gas–Liquid Interface 1 and Surface 2. The relative errors between experimental and theoretical values are 4.4%, 4.2%, 5%, 4.8%, and 4.7%, respectively. Overall, the experimental values are in great agreement with the theoretical values, the average threshold error of the six switch structures is 4.6%, and the error might be because the static threshold does not take the dynamic contact angle into account. 

**Figure 7:**
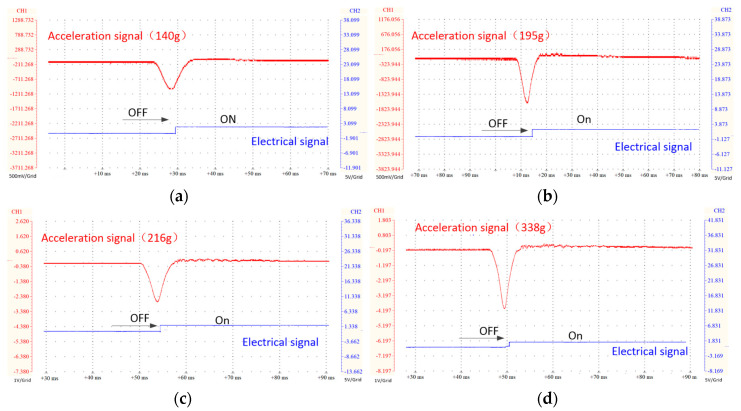
Experimental switching profile of actuation versus signal voltages. (**a**) under load of 140 g; (**b**) under load of 195 g; (**c**) under load of 216 g; (**d**) under load of 338 g.

The signal acquisition and output are carried out by a signal collector and a virtual oscilloscope. Figure 7 shows the signal response of four structural size switches. The contact electrodes of the switch are all placed at a distance of 100 µm from the valve port. In the figure, the red signal is the half-sine acceleration load and the blue signal is the electrical signal of the switch. In addition, to ensure the stability of the output signal, each switch structure was tested twice. 

As shown in Figure 7a–d, when the inertial force is greater than the threshold of the switch, the switch has a power-on signal within a short time, and the response times are 0.9, 1.1, 0.7, and 0.6 ms, respectively. After the switch electrode is connected, the liquid continues to flow under the action of the remaining inertial force until the liquid enters the equilibrium stage, and the switch remains in a stable on-state. Generally speaking, when the acceleration load is greater than the switch threshold, the switch power connection response is faster, the power connection is stable throughout the process, and no disturbance signal appears.

### Dynamic Threshold

**Figure 8:**
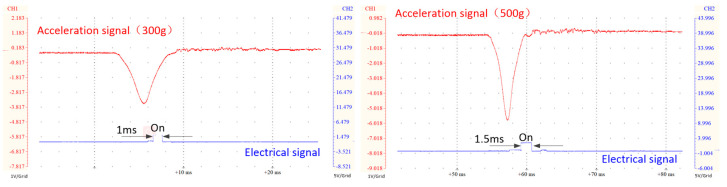
Measured switching profile of actuation versus signal voltages under different positions of electrode.

To study the dynamic threshold characteristics of the switch, for microswitches with the same structural parameters, the contact electrodes were placed at different locations for testing, and the test results are shown in Figure 8. The switch structure uses NO_3_, the electrode positions are 162 and 167 µm, and the acceleration load is 300 and 500 g, respectively. According to the test results, the response time of the switch is 1.3 and 1.9 ms, the power-on time is 1 and 1.5 ms, and the dynamic threshold ranges are 295–298 and 494–496 g, respectively. Overall, when the switch structure parameters are fixed, the response time and power-on time of the switch can be changed by changing the position of the contact electrode, and the switch can achieve a range of dynamic thresholds. In addition, based on the symmetrical structure of the U-shaped switch, the liquid levels at both ends will eventually stop at the position of the balance line (160 µm), so the switch has the functions of fast power on and off.

## 4. Conclusions

Based on the U-type microchannel, this paper studies the sensing performance of salt solution at the microscale, as well as the static threshold, dynamic threshold, and anti-high overload performance of the inertial switch. The CFD simulation results show that the U-shaped microswitch structure can distinguish the acceleration threshold. When the acceleration load is less than the switch threshold, the liquid level fails to break through the passive valve, and the liquid movement shows a certain oscillating change. However, when the acceleration load is greater than the switch threshold, the switch can achieve a rapid response to switch on. In this case, the movement of the liquid surface changes parabolically but finally stops at the position of the equilibrium line.

According to the experimental results, the U-shaped structure micro inertial switch has fast response, stable power connection, and high overload resistance. In addition, the high overload experiments show the microswitch can achieve a range of dynamic threshold by changing the placement of the contact electrode, thus can achieve rapid power on and off. Overall, the research in this paper verifies that low channel has the potential to break through the shortcomings of the low threshold value of the existing switch and the easy separation of the air–liquid surfaces. This provides an effective idea for the application of microfluidic inertial switches in high threshold and anti-high overload viscosity, where low surface tension liquids can be used as moving sensing electrodes, as well as U-type microenvironments.

## Figures and Tables

**Table 1:** The simulation model parameters.

Name	Value
R	150 µm
D/d	100/50 µm
H	100 µm
*l*/Z	50/300 µm
Brine Density	1030 kg/m^3^
Air Density	1.225 kg/m^3^
Surface Tension	0.075
Contact Angle	117°

**Table 2:** Structure parameters of the test device (µm).

No	1	2	3	4	5
H (µm)	50	50	100	100	200
D (µm)	50	100	100	200	400
H/D	1:1	1:2	1:4	1:2	1:2
Experiment value (g)	338	216	195	140	85
Theoretical value (g)	353	225	203	147	89

## References

[1] Tahirbegi I.B., Ehgartner J., Sulzer P., Zieger S., Kasjanow A., Paradiso M., Strobl M., Bouwes D., Mayr T. (2017). Fast pesticide detection inside microfluidic device with integrated optical pH, oxygen sensors and algal fluorescence. Biosens. Bioelectron..

[2] DeGraff A., Rashidi R. (2020). Ferrofluid transformer-based tilt sensor. Microsyst. Technol..

[3] Nyein H.Y.Y., Bariya M., Kivimäki L., Uusitalo S., Liaw T.S., Jansson E., Ahn C.H., Hangasky J.A., Zhao J., Lin Y. (2019). Regional and correlative sweat analysis using high-throughput microfluidic sensing patches toward decoding sweat. Sci. Adv..

[4] Freitas C.B., Moreira R.C., Tavares M.G.D.O., Coltro W.K.T. (2016). Monitoring of nitrite, nitrate, chloride and sulfate in environmental samples using electrophoresis microchips coupled with contactless conductivity detection. Talanta.

[5] Nyein H.Y.Y., Tai L.-C., Ngo Q.P., Chao M., Zhang G.B., Gao W., Bariya M., Bullock J., Kim H., Fahad H.M. (2018). A Wearable Microfluidic Sensing Patch for Dynamic Sweat Secretion Analysis. ACS Sens..

[6] Weng X., Gaur G., Neethirajan S. (2016). Rapid Detection of Food Allergens by Microfluidics ELISA-Based Optical Sensor. Biosensors.

[7] Davis L.J., Deutsch M. (2010). Surface plasmon based thermo-optic and temperature sensor for microfluidic thermometry. Rev. Sci. Instrum..

[8] Yeo J.C., Yu J., Loh K.P., Wang Z., Lim C.T. (2016). Triple-State Liquid-Based Microfluidic Tactile Sensor with High Flexibility, Durability, and Sensitivity. ACS Sens..

[9] Park U., Yoo K., Kim J. (2010). Development of a MEMS digital accelerometer (MDA) using a microscale liquid metal droplet in a microstructured photosensitive glass channel. Sens. Actuators A Phys..

[10] Michaelis S., Timme H.J., Wycisk M., Binder J. (2000). Acceleration threshold switches from an additive electroplating MEMS process. Sens. Actuators A Phys..

[11] Barbour N., Schmidt G. (2001). Inertial sensor technology trends. Sens. J. IEEE.

[12] Basu A., Hennessy R., Adams G., McGruer N. Reliability in hot switched ruthenium on ruthenium MEMS contacts. Proceedings of the 2013 IEEE 59th Holm Conference on Electrical Contacts (Holm 2013).

[13] Liu J., Ou H., Zeng R., Zhou J., Long K., Shi H., Xie Y.M. (2019). Fabrication, dynamic properties and multi-objective optimization of a metal origami tube with Miura sheets. Thin-Walled Struct..

[14] Yoo K., Kim J. A novel configurable MEMS inertial switch using microscale liquid-metal droplet. Proceedings of the IEEE 22nd International Conference on Micro Electro Mechanical Systems, MEMS 2009.

[15] Huang Y.-C., Sung W.-L., Lai W.-C., Liu C.-Y., Fang W. Design and implementation of time-delay switch triggered by inertia load. Proceedings of the 2013 IEEE 26th International Conference on Micro Electro Mechanical Systems (MEMS).

[16] Shen T., Zhang D., Huang L., Wang J. (2016). An automatic-recovery inertial switch based on a gallium-indium metal droplet. J. Micromech. Microeng..

[17] Yoo K., Park U., Kim J. (2011). Development and characterization of a novel configurable MEMS inertial switch using a microscale liquid-metal droplet in a microstructured channel. Sens. Actuators A Phys..

[18] Kuo J.C., Kuo P.H., Lai Y.T., Ma C.W., Lu S.S., Yang Y.J.J. (2013). A passive inertial switch using MWCNT–hydrogel composite with wireless interrogation capability. J. Microelectromech. Syst..

[19] Arango Y., Temiz Y., Gökçe O., Delamarche E. (2020). Electro-actuated valves and self-vented channels enable programmable flow control and monitoring in capillary-driven microfluidics. Sci. Adv..

[20] Thio T.H.G., Soroori S., Ibrahim F., Al-Faqheri W., Soin N., Kulinsky L., Madou M. (2013). Theoretical development and critical analysis of burst frequency equations for passive valves on centrifugal microfluidic platforms. Med. Biol. Eng. Comput..

[21] Khoshmanesh K., Tang S.-Y., Zhu J.Y., Schaefer S., Mitchell A., Kalantar-Zadeh K., Dickey M.D. (2017). Liquid metal enabled microfluidics. Lab Chip.

[22] Sen P., Kim C.J. (2009). A fast liquid-metal droplet microswitch using EWOD-driven contact-line sliding. J. Microelectromech. Syst..

[23] Wang Y., Duan W., Zhou C., Liu Q., Gu J., Ye H., Li M., Wang W., Ma X. (2019). Phoretic Liquid Metal Micro/Nanomotors as Intelligent Filler for Targeted Microwelding. Adv. Mater..

[24] Li J., Nie W., Liu G. (2019). Microfluidic inertial switch based on J-shape communicating vessels. Microsyst. Technol..

[25] Shen T., Li J., Huang L., Chang J., Xie J. (2019). Dynamic flow characteristics in U-type anti-high overload microfluidic inertial switch. Microfluid. Nanofluid..

